# Sol–Gel Synthesis of Silica-Based Materials with Different Percentages of PEG or PCL and High Chlorogenic Acid Content

**DOI:** 10.3390/ma12010155

**Published:** 2019-01-06

**Authors:** Elisabetta Tranquillo, Federico Barrino, Giovanni Dal Poggetto, Ignazio Blanco

**Affiliations:** 1 Department of Engineering, University of Campania “Luigi Vanvitelli”, via Roma 29, 81031 Aversa, Italy; federicobarrino92@hotmail.it; 2 Ecoricerche srl, via Principi Normanni 36, 81043 Capua Caserta, Italy; giogiodp@hotmail.it; 3 Department of Civil Engineering and Architecture and UdR-Catania Consorzio INSTM, University of Catania, Viale Andrea Doria 6, 95125 Catania, Italy; iblanco@unict.it

**Keywords:** sol–gel method, hybrid materials, antibacterial activity, chlorogenic acid, PEG, PCL, biomaterials

## Abstract

Implanted biomedical devices can induce adverse responses in the human body, which can cause failure of the implant—referred to as implant failure. Early implant failure is induced numerous factors, most importantly, infection and inflammation. Natural products are, today, one of the main sources of new drug molecules due to the development of pathogenic bacterial strains that possess resistance to more antibiotics used currently in various diseases. The aim of this work is the sol–gel synthesis of antibacterial biomedical implants. In the silica matrix, different percentages (6, 12, 24, 50 wt %) of polyethylene glycol (PEG) or poly(ε-caprolactone) (PCL) were embedded. Subsequently, the ethanol solutions with high amounts of chlorogenic acid (CGA 20 wt %) were slowly added to SiO_2_/PEG and SiO_2_/PCL sol. The interactions among different organic and inorganic phases in the hybrid materials was studied by Fourier transform infrared (FTIR) spectroscopy. Furthermore, the materials were soaked in simulated body fluid (SBF) for 21 days and the formation of a hydroxyapatite layer on their surface was evaluated by FTIR and XRD analysis. Finally, *Escherichia coli* and *Pseudomonas aeruginosa* were incubated with several hybrids, and the diameter of zone of inhibition was observed to assessment the potential antibacterial properties of the hybrids.

## 1. Introduction

Bacterial resistance to antibiotics has been observed with increasing frequency in recent decades. During implant surgery, it is possible to contract infections by pathogenic bacteria that cause serious complications for patients as these infections are often difficult to treat with antibiotic therapies that are becoming less effective [1,2,3,4,5].

In recent years, new strategies have been necessary to prevent infections associated with biomaterials [6]. In particular, natural products are, today, one of the main sources of new drug molecules due to the development of pathogenic bacterial strains that possess resistance to more antibiotics used currently in various diseases [7]. Chlorogenic acid (CGA) is present in coffee beans, as well as tea leaves, grapes, and apples. In recent years, the interest in this natural molecule has increased [8,9,10] due to its many properties that promote health, such as anticancer, anti-inflammatory, antioxidant, antidiabetic, antilipidemic, and antimicrobial activities [11]. The antimicrobial activity of CGA might be useful in the medical field to develop new antibacterial hybrid materials [12]. In this context, two different hybrid materials containing silica as inorganic matrix, with a different content (6, 12, 24, 50 wt %) of polyethylene glycol (PEG) or poly(ε-caprolactone) (PCL), were synthesized by sol–gel method; furthermore, high percentages of CGA were added in the hybrids as antibacterial drug.

The reaction products of silica-based materials can improve the biological properties, interactions between cell and material, and response to cell invasion [13,14]. The biological properties of these materials can be increased by the addition of polymers. In fact, the presence of polyethylene glycol (PEG) or polylysine improves cell adhesion and growth through increasing the hydrophilicity of materials [15,16,17,18,19], as well as PCL, which has been widely explored as a biomaterial for the controlled release of therapeutic molecules. It is a biodegradable polyester widely used in the biomedical field [20,21]. Furthermore, the silica-based materials could have, also, high antimicrobial activity when different antibacterial compounds are embedded in these materials, like antimicrobial drugs, as well as CGA or antimicrobial polymers. Gong et al. have been synthesized. Quaternary ammonium silane-functionalized methacrylate (QAMS), produced by sol–gel method, has antimicrobial activity which could help to prevent resin-based tooth filling failure due to recurrence of caries [22]. Also in the work of Makvandi et al., quaternary ammonium methacrylate-modified silica nanoparticles (QMSNs) were synthesized for the first time, and proposed as possible antimicrobial particles for free-radical, photocurable monomers [23].

One of the more interesting methods to synthesize the organic–inorganic hybrid materials at low temperature is the sol–gel technique. Two important reactions are involved; in fact, in this chemical synthesis, the precursor undergoes hydrolysis to form a colloidal suspension (sol), followed by a condensation reaction that allows “sol” evolution in a “gel” [24,25]. In the hybrid materials, when the organic and inorganic phases are bonded by hydrogen bonds, van der Waals, or ionic bonds, the materials are referred to as Class I, while, in Class II, strong chemical bonds are present (covalent or polar covalent bonds) [26]. 

Furthermore, the presence of CGA in the different hybrids allows to classify the materials as “green hybrids”, like reported by Unterlass [27]. In fact, when the hybrids are synthesized using at least one green step or a component of green origin is incorporated, these materials are defined as “green hybrids”.

The purpose of this work is the synthesis of new hybrids with antibacterial activity depending on the incorporated CGA but, also, depending on the different types of polymers and their percentage. The presence of the different interactions in the hybrid materials was studied with Fourier transform infrared (FTIR) spectroscopy. In order to evaluate the hybrids’ bioactivity, FTIR spectroscopy was used to observe the typical peaks of hydroxyapatite on the materials’ surface, after soaking in simulated body fluid (SBF), and XRD was carried out to confirm the FTIR results. Finally, two different bacteria, *E. coli* and *P. aeruginosa*, were incubated with the organic–inorganic hybrid materials to investigate potential antibacterial activity.

## 2. Materials and Methods 

### 2.1. Sol–Gel Synthesis of the Hybrid Materials

The sol–gel method was used to synthesize the different hybrid materials. The precursor of silica matrix, tetraethyl orthosilicate (TEOS; Si(OC_2_H_5_)_4_; Sigma-Aldrich, Darmstadt, Germany), was added to a solution of 99.8% ethanol (EtOH, Sigma-Aldrich, Darmstadt, Germany), HNO_3_ (≥65%, Sigma-Aldrich, Darmstadt, Germany), and distilled water. The gel material was obtained by hydrolysis and condensation reactions, therefore, nitric acid was used to favor the kinetics of these reactions. The molar ratios of solution were EtOH/TEOS = 6.2, TEOS/HNO_3_ = 1.7, and H_2_O/TEOS = 6. 

In order to prepare the SiO_2_/PEG/CGA hybrids or SiO_2_/PCL/CGA hybrids, different amounts of PEG, dissolved in ethanol, or PCL, dissolved chloroform, were added in silica sol, with the same percentages (6, 12, 24, 50 wt %), separately [15,18].

Afterwards, the ethanol solutions with high amounts of CGA (20 wt %) (95%, Sigma Aldrich) were slowly added to the SiO_2_/PEG or SiO_2_/PCL solutions. The remaining solvents in the wet gels were removed by incubating in an oven at 40 °C for 24 h.

**Figure 1:**
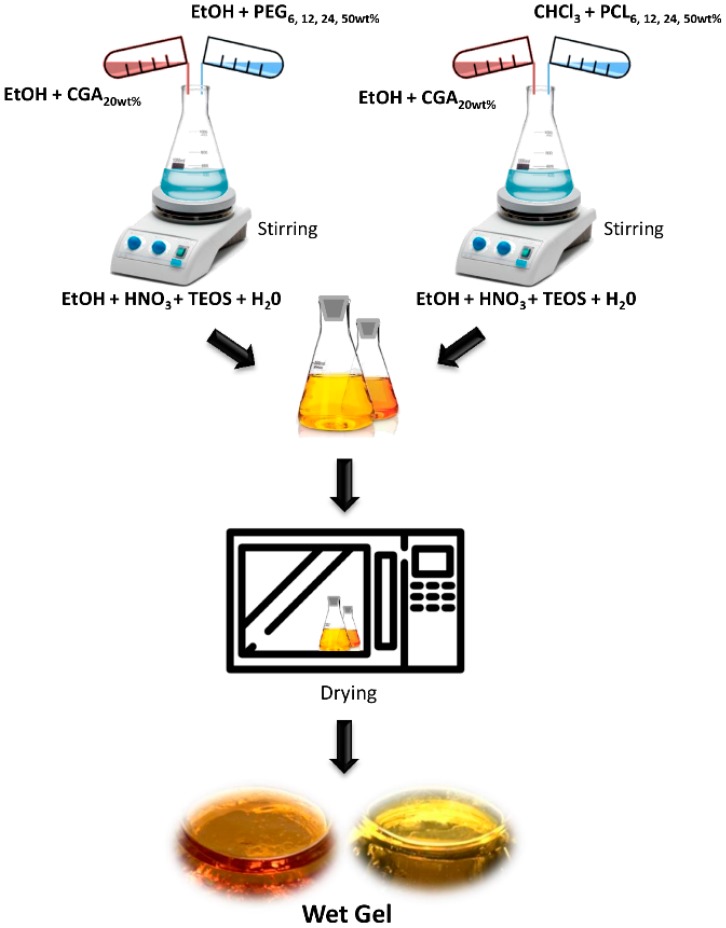
Flow chart of sol–gel synthesis.

The Figure 1 shows the flow chart of the hybrid synthesis.

### 2.2. SEM and FTIR Analysis of the Hybrid Materials

Prestige 21 Shimadzu (Kyoto, Japan) FTIR instrument equipped with a DTGS detector was used to evaluate the different interactions among the inorganic and organic components of the hybrids.

The samples were analyzed in KBr pelletized disks containing about 2 mg of each sample and about 198 mg of KBr. The disks with a diameter of 13 mm, a thickness of 2 mm, were obtained by pressing the sample powder and KBr into a cylindrical holder using a Specac manual hydraulic press. Analysis was performed with Fourier transform infrared (FTIR) transmittance over a wavenumber range of 4000–400 cm^−1^ with resolution of 4 cm^−1^ (45 scans). The FTIR spectra were processed by Prestige software (IR solution). The surface morphology of the hybrid materials was investigated by scanning electron microscopy analysis performed using AURIGA Zeiss High Resolution Field Emission equipment (HR-FESEM, JEOL JSM-7000F, Zeiss, Kyoto, Japan).

### 2.3. Bioactivity Test

The bioactivity of the hybrid materials was evaluated using simulated body fluid (SBF) [28]. The dried gels were grinded in a mortar to obtain powders, and they were soaked in SBF for 21 days at 37 °C. The SBF solution contained an ion concentration almost equal to those of human blood plasma (Table 1). The SBF was prepared by dissolving reagent grade chemicals NaCl, NaHCO_3_, KCl, MgCl_2_, 1 M HCl, CaCl_2_·6H_2_O, and Na_2_SO_4_ (Sigma-Aldrich, St. Louis, MO, USA). The pH of the buffer was adjusted to pH 7.4 using 1 M HCl.

Every two days, the solution was replaced to avoid depletion of ionic species in the SBF caused by nucleation of biominerals on the samples. After these days of exposure, the samples were dried in a glass desiccator and, afterwards, the formation of apatite layer on sample surface was evaluated by FTIR analysis and XRD analysis. XRD analysis was carried out in the range of 2θ from 20 to 70° using a Philips 139 diffractometer (Philips, Amsterdam, Netherlands) equipped with a PW 1830 generator, tungsten lamp, and Cu anode, where the source 140 of X-ray is given by a Cu-Kα radiation (λ = 0.15418 nm).

### 2.4. Antibacterial Activity

The influence of materials on the microbial growth of *Escherichia coli* (ATCC 25922) and *Pseudomonas aeruginosa* (ATCC 10145) was assessed.

*E. coli* was incubated in TBX medium (tryptone bile X-gluc) (Liofilchem, Italy), while *Pseudomonas aeruginosa* in Pseudomonas CN agar (Liofilchem, Italy). The bacterial cell suspension of 10 × 10^5^ cfu/mL was obtained by dilution in distilled water. Afterwards, *E. coli* was incubated in presence of the different materials for 24 h at 44 °C, while the *Pseudomonas aeruginosa* was incubated for 48 h at 36 °C.

The diameter of the inhibition halo (ID) was measured to evaluate microbial growth. The results were obtained on samples analyzed three times and used to determine the mean standard (SD) deviation of measurements.

## 3. Results

### 3.1. SEM and FTIR Analysis of the Hybrid Materials

All hybrids containing PEG as well as PCL, with a high amount of CGA, are transparent, glassy, yellowish, and reddish, as a function of PCL and PEG, respectively, but, also, affected by the presence of CGA. Representative samples are reported in Figure 1.

**Figure 2:**
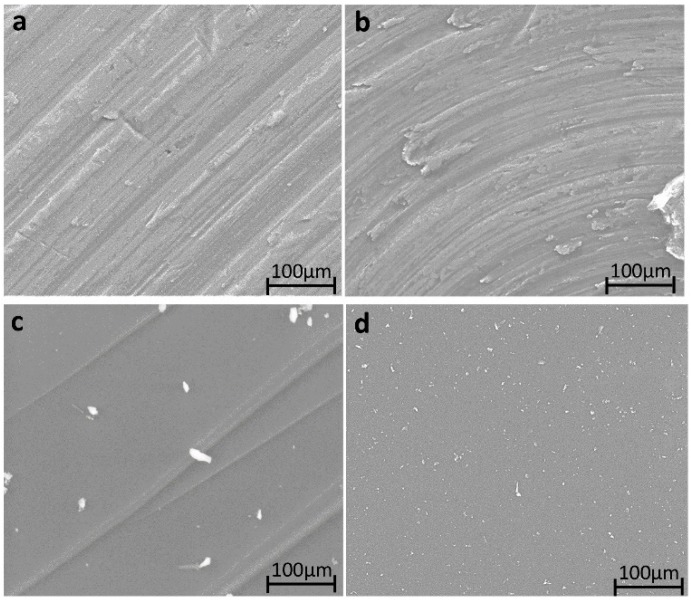
SEM micrograph of (**a**) SiO_2_/PEG_50 wt %_; (**b**) SiO_2_/PCL_50 wt %_; (**c**) SiO_2_/PEG_50 wt %_/CGA_20 wt %_; (**d**) SiO_2_/PCL_50 wt %_/CGA_20 wt %._

The microstructure of the hybrids with different percentages of PEG or PCL, and the hybrid materials contain the two polymers and CGA, have been studied by SEM (Figure 2). In the surface morphology of the samples, there is no appreciable difference among the all materials. The results suggest that the chlorogenic acid embedded in the materials does not affect the morphology of SiO_2_/PEG and SiO_2_/PCL hybrids, respectively.

**Figure 3:**
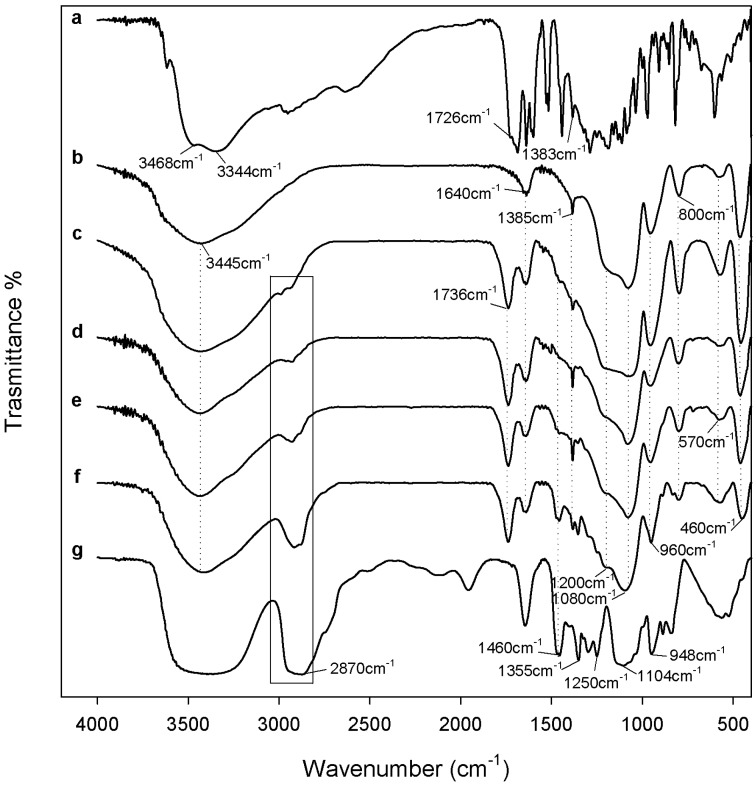
Representative FTIR spectra (**a**) CGA; (**b**) SiO_2_; (**c**) SiO_2_/PEG_6wt %_/CGA_20wt %_; (**d**) SiO_2_/PEG_12wt %_/CGA_20wt %_; (**e**) SiO_2_/PEG_24wt %_/CGA_20wt %_; (**f**) SiO_2_/PEG_50wt %_/CGA_20wt %_; (**g**) PEG.

**Figure 4:**
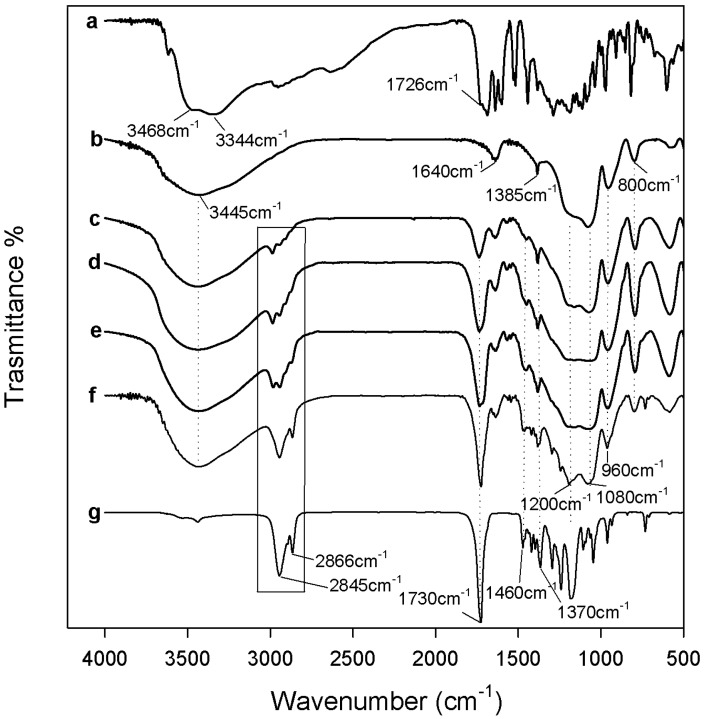
Representative FTIR spectra (**a**) CGA; (**b**) SiO_2_; (**c**) SiO_2_/PCL_6 wt %_/CGA_20 wt %_; (**d**) SiO_2_/PCL_12 wt %_/CGA_20 wt %_; (**e**) SiO_2_/PCL_24 wt %_/CGA_20 wt %_; (**f**) SiO_2_/PCL_50 wt %_/CGA_20 wt %_; (**g**) PCL.

In Figure 3 and Figure 4 are reported the FTIR spectra of different hybrids that were compared with pure PEG, PCL, and pure silica, to identify their interaction. In all spectra, the typical band of the silica matrix is clearly visible [29,30]. The peaks recorded at 1080, 1200, and 800 cm^−1^ were due to the asymmetric and symmetric Si–O stretching vibrations. The bending vibrations of Si–O–Si bonds and Si–OH bond vibrations were attributed to the peaks at 460 cm^−1^ and 960 cm^−1^, respectively [31]. Furthermore, in all hybrid spectra, regardless of the content of the two different polymers and CGA, the intensity band at 580 cm^−1^ is visible, caused by residual four-membered siloxane rings in the silica network [29,30,32]. Also, the residual nitrate anions at 1382 cm^−1^ with a sharp N–O stretching band [33] is observed because, during the synthesis procedure, the HNO_3_ catalyst was used. 

Furthermore, the bands at 3445 cm^−1^ and 1640 cm^−1^, due to –OH stretching and bending vibrations, change position and shape for the formation of hydrogen bonded solvent molecules (H_2_O) and hydrogen-bonded –OH groups related to Si atoms [34]. 

The typical peaks of natural compound (CGA) are visible in all hybrid spectra, with similar intensity. In the spectrum of pure CGA (Figure 3 and Figure 4 curve a), the stretching C=O vibration band is observed at 1726 cm^−1^. Instead, the displacement of this peak is visible in all of the hybrid spectra, independent of the polymer type incorporated, due to the formation of H-bonds with the SiO_2_ inorganic matrix [35]. Furthermore, the presence of intense signals of the phenyl ring and C–O–C bonds caused the different shape and a broadening of the strong SiO_2_ band at 1080 cm^−1^, and the increase of the intensity of the shoulder at 1200 cm^−1^ (Figure 3 and Figure 4 curve a), which is also due to the interactions with the two different polymers [36]. 

Comparing Figure 3 and Figure 4, it is possible to observe some different peaks due to several polymers incorporated in the hybrids. When different amounts of PEG were added in the materials (Figure 3), the bands of methylene C–H stretching and bending at 2930–2870 cm^−1^ and 1454 cm^−1^ are clearly detectible in all spectra, furthermore, its intensity increased with increasing polymer amount [37]. The characteristic C–O stretching band at 1250 cm^−1^ is evident only when a high amount of PEG was added in the hybrid [38]. Indeed, the presence of PCL in the materials (Figure 4) determines the appearance of typical peaks not visible in Figure 3. The asymmetric and symmetric stretching of polymer CH_2_ groups correspond to the bands at 2845 and 2866 cm^−1^, while the peaks at 1460 cm^−1^ and 1370 cm^−1^ are due to their bending modes. Moreover, when a high amount of PCL was added in the hybrid (Figure 4 curve f), it is possible to observe a different shape of the typical silica band at 1080 cm^−1^ caused by the asymmetric O–C–O stretching of the polymer ester group and, also, the peak at 1730 is attributable to the CO stretching vibration of the ester group [39]. 

In all hybrid spectra (Figure 4), the peak at 1736 cm^−1^, that is due to the stretching C=O vibration of CGA, changes its intensity, despite that the concentration of CGA in the hybrid is the same and, in fact, this effect is only due to the different amount of PCL. The intensity of the characteristic PCL and PEG bands seemed strongly affected by the polymer content. The formation of the interactions between all hybrid components are suggested by the displacement of the silica and polymers bands at a lower wavenumber. The chlorogenic acid incorporated in the hybrids does not affect the structure and interactions of SiO_2_/PEG and SiO_2_/PCL hybrid materials, respectively. Therefore, all components are bonded by hydrogen bonds, that are very important in the hybrids structure.

### 3.2. Bioactivity Test

Kokubo’s test [28] was used to evaluate the bioactivity of hybrids as a function of CGA and polymer content. Kokubo and his colleagues [28,40,41] have proposed that one of the important requirements for a good biomaterial is the formation of apatite on its surface after implantation in the human body. SBF solution, containing an ion concentration similar to that of human blood plasma, can be used to produce the apatite layer; therefore, the standard protocol introduced by Kokubo et al. has been followed [28]. The different hybrids were soaked in SBF solution for 21days and, after this exposure time, the presence of the hydroxyapatite on the surfaces of all samples was detected by FTIR and XRD. 

**Figure 5:**
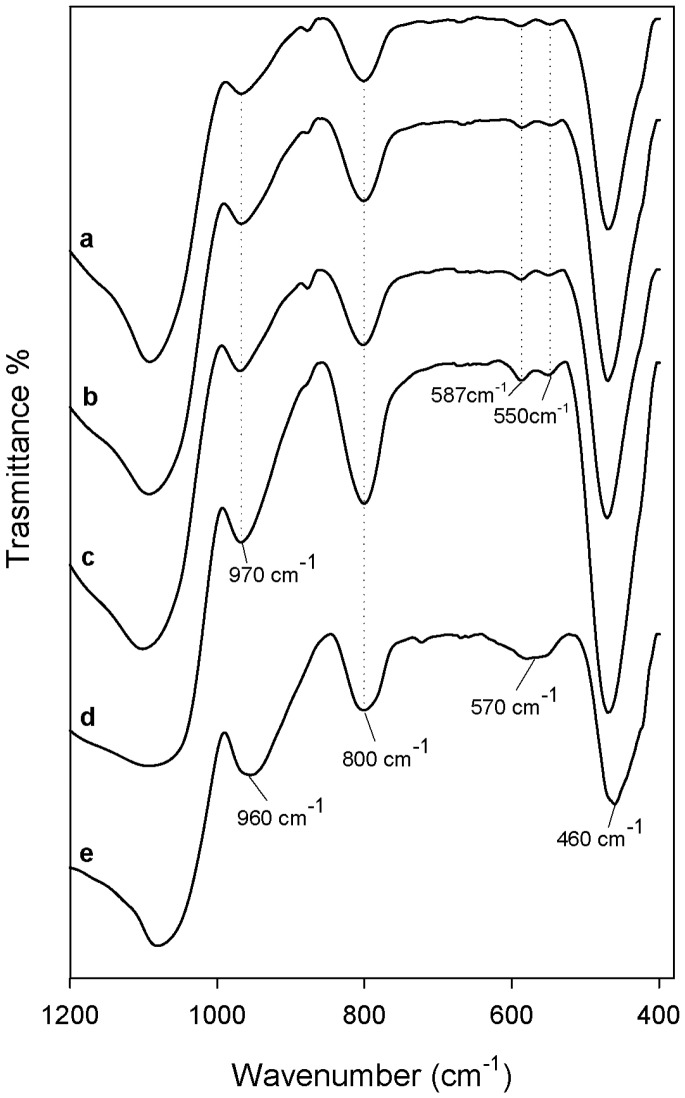
Representative FTIR spectra of (**a**) SiO_2_/PEG_6 wt %_/CGA_20 wt %_; (**b**) SiO_2_/PEG_12 wt %_/CGA_20 wt %_; (**c**) SiO_2_/PEG_24 wt %_/CGA_20 wt %_; (**d**) SiO_2_/PEG_50 wt %_/CGA_20 wt %_ after 21 days of exposure to SBF; and (**e**) SiO_2_/PEG_12 wt %_/CGA_20 wt %_ before soaking in SBF.

**Figure 6:**
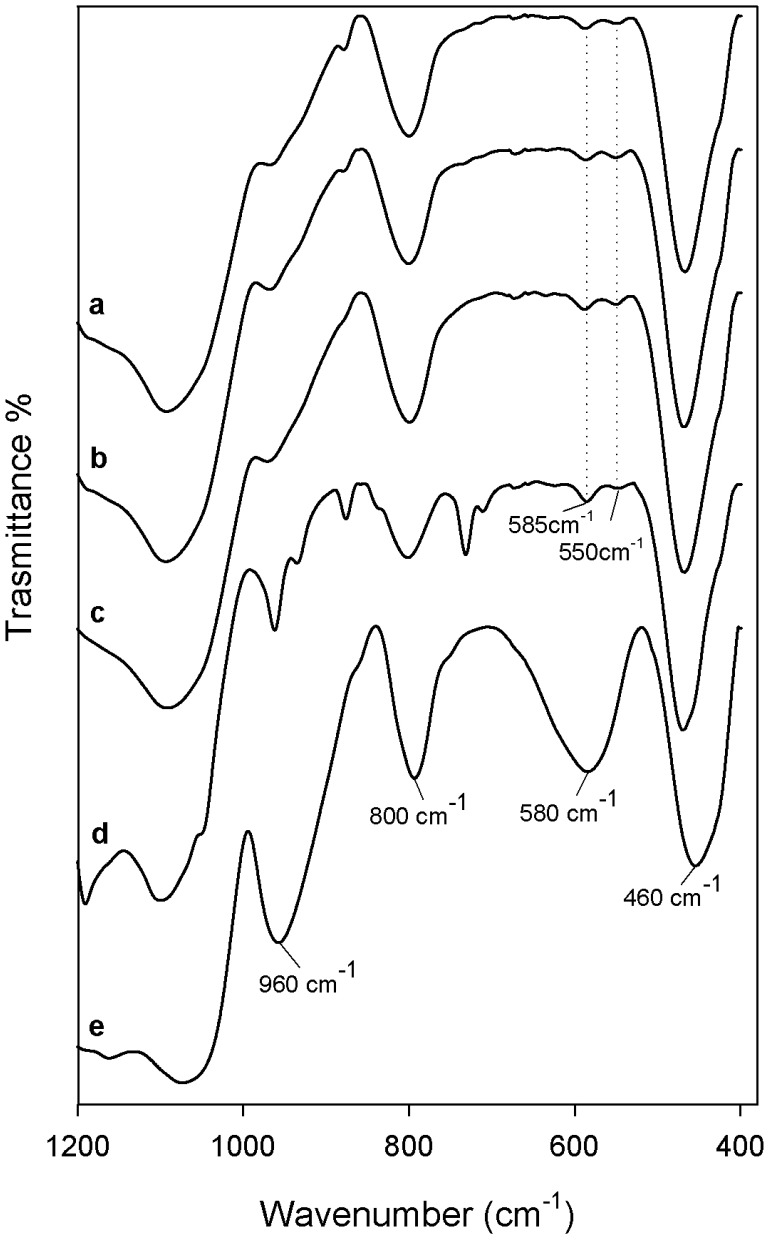
Representative FTIR spectra of (**a**) SiO_2_/PCL_6 wt %_/CGA_20 wt %_; (**b**) SiO_2_/PCL_12 wt %_/CGA_20 wt %_; (**c**) SiO_2_/PCL_24 wt %_/CGA_20 wt %_; (**d**) SiO_2_/PCL_50 wt %_/CGA_20 wt %_ after 21 days of exposure to SBF; and (**e**) SiO_2_/PCL_12 wt %_/CGA_20 wt %_ before soaking in SBF.

Figure 5 and Figure 6 show the FTIR spectra of both materials containing PEG and PCL with high amounts of CGA, respectively. 

In all hybrid spectra, the split of the bands at 570 cm^−1^ and 580 cm^−1^ in the hybrids, with PEG and PCL, respectively, into two new ones was observed. In particular, in Figure 5 (curve a–d), bands at 587 cm^−1^ and 550 cm^−1^ were detected while, in Figure 6 (curve a–d), the same bands at 585 cm^−1^ and 550 cm^−1^ are visible. These peaks suggest the stretching of the –OH groups of hydroxyapatite and the vibrations of the PO_4_^3−^ groups caused by the formation of hydroxyapatite precipitate [40,41,42,43]. 

The displacement of Si–OH band, from 955 cm^−1^ to 960 cm^−1^, is due to the interaction of the hydroxyapatite layer with the –OH groups of the silica matrix.

**Figure 7:**
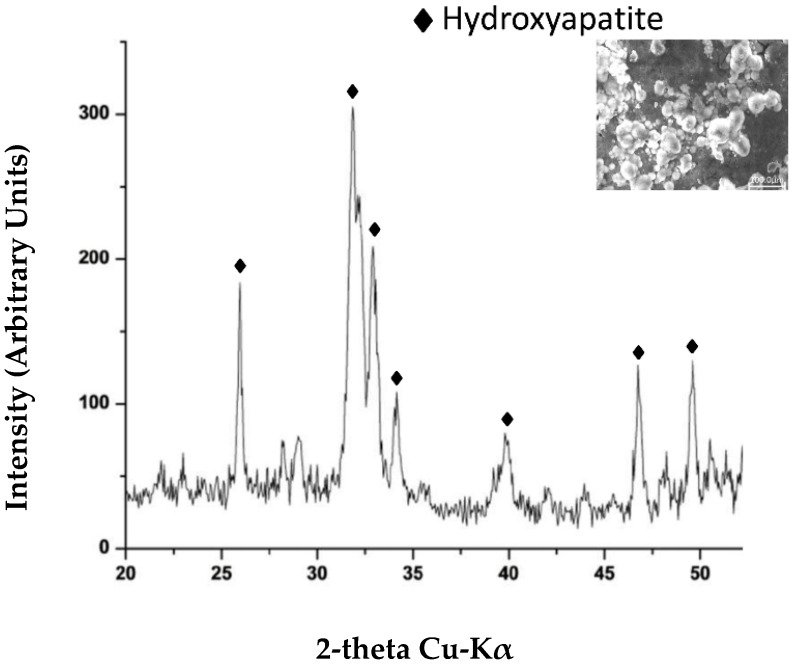
A representative XRD of SiO_2_/PEG_50 wt %_/CGA_20 wt %_ soaked in SBF solution for 21 days.

The presence of hydroxyapatite on the materials surfaces is also confirmed by XRD measurement (Figure 7), in fact, the intense peaks of hydroxyapatite crystalline are visible in all materials, and the peaks obtained were similar with the phases found in the ICDD database. The main (h k l) indices for hydroxyapatite are (002), (211), (300), (202), (310), (002), (222), and (213), regardless of the content of the two different polymers and CGA, after 21 days in SBF. 

The hydroxyapatite layer (Ca_10_(PO_4_)_6_(OH)_2_) occurs when the Ca^2+^ ions, present in the SBF, interact with the –OH groups on the silica matrix and, subsequently, with the negative charge of the phosphate ions [44].

### 3.3. Antibacterial Activity

**Figure 8:**
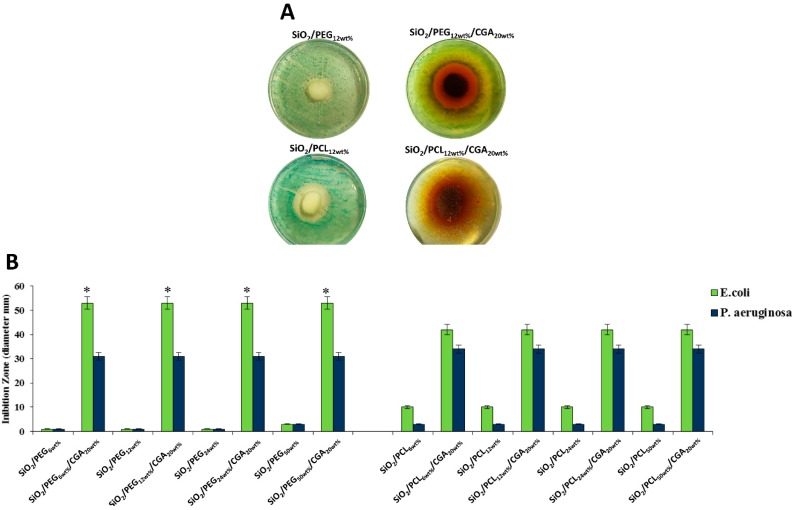
(**A**) Representative inhibition halo (ID) of *E. coli* with SiO_2_/PEG_12 wt %_, SiO_2_/PEG_12 wt %_/CGA_20 wt %_, SiO_2_/PCL_12 wt %_, and SiO_2_/PCL_12 wt %_/CGA_20 wt %_; (**B**) The diameter (mm) of inhibition zone of all materials. Values are the mean SD of measurements carried out on samples analyzed three times. The means and SD are shown. * *p* < 0.05 vs. the bacteria control treated with hybrids without CGA or vs. the bacteria treated with hybrids containing CGA.

The antibacterial properties were evaluated using two different bacteria: *E. coli* and *P. aeruginosa*. Both bacteria were incubated in presence of the 100 mg of hybrid materials. In Figure 8A is reported a representative image of *E. coli* inhibition zones. The results of the diameter of zone of inhibition (Figure 8B) of all hybrids suggested that when both bacteria were inoculated with the materials containing different amount of PEG without CGA, the inhibition of bacterial growth is not observed compared to the materials with only different percentages of PCL; in fact, in SiO_2_/PCL, a mild antibacterial activity is visible.

However, the hybrids with high amount of CGA, independently of the polymer type incorporated, exhibited a strong activity against *E. coli* compared to *P. aeruginosa*. The antibacterial activity observed is due to the presence of CGA in the hybrids. In the literature are reported many works about the molecular mechanism of this natural compound [45,46,47]. The damage of the bacterial cell membrane could be caused by reactive oxygen species (ROS) depletion due to CGA in the different materials [11]. Therefore, in the cells, when levels of ROS decrease, numerous signaling pathways are affected, allowing inhibition of bacterial growth [48]. 

Finally, the diameter of the zone of inhibition of SiO_2_/PEG/CGA and SiO_2_/PCL/CGA were compared. When *E. coli* were treated with the hybrids containing PEG, better antibacterial activity was observed (Figure 8).

Furthermore, when *E. coli* and *P. aeruginosa* were incubated with the hybrids containing CGA, it was possible to observe a different color of the medium in the zone of inhibition around the materials, compared to the medium of both bacteria treated with the hybrids without CGA (Figure 8A). This effect is probably due to the release of CGA from the different materials, causing the inhibition of bacterial growth around the hybrids.

## 4. Conclusions

The hybrid materials containing PEG or PCL and a high amount of CGA were synthesized by sol–gel method. The interactions between the components in the hybrids were evaluated by FTIR analysis. The spectra suggested that the organic and inorganic phases are linked through hydrogen bonds. Furthermore, the standard protocol introduced by Kokubo et al. [28] was used for in vitro bioactivity assessment of the different materials, and the results suggested that the bioactivity of the silica matrix was not affected by the presence of PEG and PCL, as well as the CGA. Furthermore, independently of the polymer types incorporated in the hybrid, it was possible to observe a strong activity against *E. coli* compared to *P. aeruginosa*, in particular, with SiO_2_/PEG/CGA; this effect is due to the presence of a high amount of CGA. In conclusion, the materials synthesized can be considered as potential antibacterial hybrids which could be used as biomedical implants.

## Figures and Tables

**Table 1:** Simulated body fluid (SBF) composition.

Ion	Concentration/mol m^3^	
	SBF	Human Blood Plasma
Na^+^	142.0	142.0
K^+^	5.0	5.0
Mg^2+^	1.5	1.5
Ca^2+^	2.5	2.5
Cl^−^	147.8	103.0
HCO_3_^−^	4.2	27.0
HPO_4_^2−^	1.0	1.0
SO_4_^2−^	0.5	0.5
